# Integrating Ataxia Evaluation into Tumor-Induced Hearing Loss Model to Comprehensively Study NF2-Related Schwannomatosis

**DOI:** 10.3390/cancers16111961

**Published:** 2024-05-22

**Authors:** Simeng Lu, Zhenzhen Yin, Jie Chen, Limeng Wu, Yao Sun, Xing Gao, Peigen Huang, Justin T. Jordan, Scott R. Plotkin, Lei Xu

**Affiliations:** 1 Edwin L. Steele Laboratories, Department of Radiation Oncology, Massachusetts General Hospital and Harvard Medical School, Boston, MA 02114, USA; 2 Department of Oral and Maxillofacial Surgery, Xiangya Hospital, Central South University, Changsha 410008, China; 3 Department of Stomatology, Peking Union Medical College Hospital, Chinese Academy of Medical Sciences and Peking Union Medical College, Beijing 100073, China; 4 Department of Radiation Oncology, Tianjin Medical University Cancer Institute and Hospital, National Clinical Research Center for Cancer, Tianjin Clinical Research for Cancer, Key Laboratory of Cancer Prevention and Therapy, Tianjin 300060, China; 5 Stephen E. and Catherine Pappas Center for Neuro-Oncology, Department of Neurology and Cancer Center, Massachusetts General Hospital, Boston, MA 02114, USA

**Keywords:** hearing loss, ataxia testing, motor function, brain tumor, mouse model

## Abstract

**Simple Summary:**

The hallmark of NF2 is bilateral vestibular schwannomas, which progressively enlarge, leading to sensorineural hearing loss, tinnitus, facial weakness, and pain that translates to social impairment and clinical depression. To better understand disease progression and characterize treatment response, we developed a panel of five tests suitable for the mouse vestibular schwannoma model and investigated how tumor growth and treatment affect gait, coordination, and motor function. These methods, paired with hearing tests, enable a comprehensive evaluation of tumor-induced neurological deficits and facilitate the assessment of the effectiveness of novel therapeutics to improve NF2 treatments.

**Abstract:**

NF2-related Schwannomatosis (NF2-SWN) is a disease that needs new solutions. The hallmark of NF2-SWN, a dominantly inherited neoplasia syndrome, is bilateral vestibular schwannomas (VSs), which progressively enlarge, leading to sensorineural hearing loss, tinnitus, facial weakness, and pain that translates to social impairment and clinical depression. Standard treatments for growing VSs include surgery and radiation therapy (RT); however, both carry the risk of further nerve damage that can result in deafness and facial palsy. The resultant suffering and debility, in combination with the paucity of therapeutic options, make the effective treatment of NF2-SWN a major unmet medical need. A better understanding of these mechanisms is essential to developing novel therapeutic targets to control tumor growth and improve patients’ quality of life. Previously, we developed the first orthotopic cerebellopontine angle mouse model of VSs, which faithfully mimics tumor-induced hearing loss. In this model, we observed that mice exhibit symptoms of ataxia and vestibular dysfunction. Therefore, we further developed a panel of five tests suitable for the mouse VS model and investigated how tumor growth and treatment affect gait, coordination, and motor function. Using this panel of ataxia tests, we demonstrated that both ataxia and motor function deteriorated concomitantly with tumor progression. We further demonstrated that (i) treatment with anti-VEGF resulted in tumor size reduction, mitigated ataxia, and improved rotarod performance; (ii) treatment with crizotinib stabilized tumor growth and led to improvements in both ataxia and rotarod performance; and (iii) treatment with losartan did not impact tumor growth nor ameliorate ataxia or motor function. Our studies demonstrated that these methods, paired with hearing tests, enable a comprehensive evaluation of tumor-induced neurological deficits and facilitate the assessment of the effectiveness of novel therapeutics to improve NF2 treatments.

## 1. Introduction

NF2-related Schwannomatosis (NF2-SWN) is a dominantly inherited neoplasia syndrome resulting from a germline mutation of the *NF2* tumor suppressor gene [1]. NF2-SWN has an incidence of 1 in 25,000 persons and a penetrance of nearly 100% [1]. The hallmark of NF2-SWN is bilateral vestibular schwannomas (VSs), which progressively enlarge, leading to sensorineural hearing loss, as well as other neurological symptoms, including tinnitus, facial weakness, impaired balance, and pain [2]. These neurological symptoms can translate to social impairment and clinical depression [2]. VSs can also cause brainstem compression, resulting in severe morbidity and mortality [3]. Standard treatments for growing VSs include surgery and radiation therapy; however, both carry the risk of further damaging the nerve [4,5,6]. The resultant suffering and debility, in combination with the paucity of therapeutic options, make the effective treatment of NF2-SWN a major unmet medical need.

The goal of our research is to find novel treatments that can simultaneously control tumor growth and alleviate neurological symptoms to improve patient quality of life. In order to better understand the biology of VS tumor progression and tumor-induced hearing loss, we previously established an orthotopic VS mouse model by directly implanting tumor cells into the cerebellopontine angle (CPA) in mice and reproduced tumor-induced hearing loss as observed in VS patients [7]. Using this model to explore novel potential therapeutic targets, we discovered that (i) cMET inhibitors can sensitize VSs to radiation therapy without any adverse effects on hearing function [8] and that (ii) losartan, an FDA-approved anti-hypertensive drug, by blocking the inflammatory angiotensin signaling reduces neuroinflammation and prevents tumor-induced hearing loss [9]. During these studies, in addition to hearing loss, we observed that mice also exhibited symptoms of ataxia and incoordination. Patients with bilateral VSs may experience damage to the vestibular nerve and the vestibule [10], leading to debilitating vestibular deficits such as impaired balance, ataxia, and muscle weakness [11]. A recent study characterized vestibular dysfunction in NF2 patients using a panel of tests [12]. They demonstrated that vestibular tests evaluating dizziness, gait, vestibular–ocular reflex, and balance could reflect disease progression and treatment efficacy. This study prompted us to establish vestibular tests suitable for the mouse VS model and investigate how tumor growth and treatment affect gait and coordination in mouse VS models.

Here, we used a panel of five tests to characterize how tumor growth and treatments affect gait, coordination, and motor function in VS models. We demonstrated that both ataxia and motor function deteriorated concomitantly with tumor progression. Treatment with anti-VEGF resulted in tumor size reduction, mitigated ataxia, and improved rotarod performance. Similarly, treatment with crizotinib stabilized tumor growth and led to improvements in both ataxia and rotarod performance. Conversely, treatment with losartan did not impact tumor growth nor ameliorate ataxia or motor function. Our studies demonstrated that these methods, paired with hearing tests, enable a comprehensive evaluation of tumor-induced neurological deficits and facilitate the assessment of the effectiveness of novel therapeutics to improve NF2 treatments.

## 2. Materials and Methods

### 2.1. Cell Lines and Reagents

Mouse *Nf2*^−/−^ Schwann cells were maintained in 10% Schwann cell medium containing Schwann cell growth supplement (SCGS, ScienCell, Carlsbad, CA, USA) [13]. Losartan potassium was obtained from TCI America (Boston, MA, USA). Anti-mouse and human VEGF antibody (B20-4.1.1) was provided by Genentech under a material transfer agreement [14].

### 2.2. Animal Models

Male and female syngeneic immune-competent FVB/C57BL/6 mice (10–12 weeks of age) were used. We breed and maintain our mice at the Gnotobiotic Cox 7 mouse colony at the Massachusetts General Hospital (MGH). All animal procedures were performed following the guidelines of the Public Health Service Policy on Humane Care of Laboratory Animals and approved by the Institutional Animal Care and Use Committee of the MGH (Animal Welfare Assurance Number: D16-00461, 2024). We used two xenograft models in our study. In both models, 1 × 10^5^ mouse *Nf2*^−/−^ cells were implanted per mouse.

- Cerebellopontine angle (CPA) model: To recapitulate the intracranial microenvironment of VSs, we implanted tumor cells and injected them into the CPA region of the right hemisphere [7]. The syringe was fixed on the stereotactic device at an angle of 15 degrees perpendicular to the work surface. The needle was moved 2.2 mm to the right to the confluence of the sinuses, then moved backward to the transverse sinus, and lastly moved 0.5 mm to the dorsal side. The syringe was then lowered until it touched the surface, moved 3.7 mm downward, and retracted 1 mm, and then 1 microliter of tumor cell suspension was injected slowly over 45–60 s. Tumor formation in the correct anatomic location was confirmed by small animal MRI and H&E staining [7].
- Sciatic nerve model: to reproduce the microenvironment of peripheral schwannomas, we implanted tumor cells into the mouse sciatic nerve [8,14]. Three microliters of tumor cell suspension were injected slowly (over 45–60 s) under the sciatic nerve sheath using a Hamilton syringe to prevent leakage.

### 2.3. Treatment Protocols

In the CPA model, when the plasma Gluc level reached 2 × 10^6^ RLU, and in the sciatic nerve model, when the tumor reached 4 mm in diameter, mice were randomized into groups to receive the following treatment:

- Losartan treatment: Losartan (40 mg/kg) was administered by oral gavage daily; treatment was continued until the mice in the control group became moribund [15,16].
- B20 treatment: B20 (5 mg/kg) was administered *i.p.* once a week; treatment was continued until the tumors reached 1 cm in diameter [14].

### 2.4. Measurement of Tumor Growth

*Nf2*^−/−^ cells were infected with lentivirus-encoding secretive Gaussia luciferase reporter gene (Gluc). The measurement of plasma Gluc was performed as previously described [7,8]. Briefly, 10 μL of whole blood were collected from a slight nick on the tail veils and mixed with 5 μL 50 mM EDTA immediately to avoid clotting. Blood samples were transferred to a 96-well plate and Gluc activity was measured using a plate luminometer (GloMax^®^ 96 Microplate Luminometer, Promega, Madison, WI, USA). The luminometer was set to automatically inject 100 µL of 100 mM coelenterazine (CTZ, Nanolight, Pinetop, AZ, USA) in PBS and photon counts were acquired for 10 s.

### 2.5. Gait and Coordination Tests

We tested the severity of gait and coordination symptoms using a group of tests. These tests are adapted from previously published phenotype assessments used in mouse models of Huntington’s disease, spinocerebellar ataxias, and spinobulbar muscular atrophy [17].

- The ledge test was carried out by lifting the mouse, placing it on the cage’s ledge, and observing it as it walked along the cage ledge. Score 3: Normal mice typically walked along the ledge and attempted to descend back into the cage using their paws. Based on the time that the mouse stayed on the ledge, a ledge test score was given. Score 2: The mouse stayed on the ledge >10 s but lost its footing and fell off the ledge. Score 1: The mouse stayed on the ledge <10 s but lost its footing and fell off the ledge. Score 0: The mouse could not stay on the ledge for longer than 3 s, or shook and refused to move at all.
- A hind-limb clasping test was performed to assess whether the mouse clasped its forelimbs and hindlimbs to its body or splayed its limbs when suspended by its tail. The mouse was lifted clear of all surrounding objects by grasping its tail near its base. The hindlimb position was observed for 10 s and recorded. Score 3: Normal mice typically splayed out their hindlimbs. Score 2: One hindlimb was retracted to the belly. Score 1: Both hindlimbs were partially retracted. Score 0: Both hindlimbs were entirely retracted and touched the belly.
- Gait is a measure of coordination and muscle function. To obtain footprints, hind- and forepaws were dipped in black ink. Then, the animals were allowed to walk, and footprint patterns made on white paper lining the floor were obtained. Score 3: In normal mice, there was no limp and the belly did not touch the ground. Score 2: The mouse walked slowly, with a slight limp. Score 1: The mouse showed severe limping and lost coordination. Score 0: The mouse had difficulty moving forward, dragged its body along the ground, and could not walk a straight line.
- Kyphosis refers to an abnormally curved spine. For this test, the mice were removed from their cages and placed on a flat surface. We assessed the characteristic dorsal curvature of the spine. Score 3: Normal mice straightened their spines as they walked. Score 2: The mouse could straighten its spine and exhibited a mild hunchback. Score 1: The mouse could not straighten its spine and exhibited a mild hunchback. Score 0: the mouse exhibited a pronounced hunchback.

### 2.6. Rotarod Test

Rotarod performance was evaluated using an automated rotarod (Rotamex 4/8 4-Lane Treadmill Shock Grid; Columbus Instruments, Livoniz, MI, USA). The rotarod test was performed on animals bearing size-matched tumors. Tumor size was measured every 3 days by caliper; when the tumor size reached ~3 mm in diameter, the mice were trained on the rotarod every day for 3 days. Three days later, we performed the rotarod test. Three tests were performed for each animal; each test began with a 30 s acclimation period at 4 rpm followed by an acceleration of 4 rpm every 60 s to a maximum of 10 min and 40 rpm. The amount of time that elapsed before the mouse fell off was recorded as the rotarod endurance. To avoid heterogeneity between animals, the average time to fall from the rotating cylinder was normalized to the value from each mouse on the first day and presented as normalized rotarod endurance [14].

### 2.7. Statistical Analysis

Efforts were made to minimize animal suffering and to reduce the number of animals used according to the 3R principles. The primary endpoints of our study are to assess the ataxia score and rotarod performance and compare the gait and coordination function between different treatments. We determined whether growth curves significantly differed from each other by log-transforming the data, fitting a linear regression to each growth curve, and comparing the slopes of the regression lines (using an equivalent of ANOVA). Significant differences between the two groups were analyzed using the Student’s *t*-test (two-tailed) or Mann–Whitney *U* test (two-tailed). All calculations were carried out using the GraphPad Prism Software 6.0 and Microsoft Excel Software 2010.

## 3. Results

### 3.1. Establish a Panel of Functional Tests to Evaluate the Severity of Gait and Coordination Symptoms in the VS Mouse Model

To evaluate ataxia and incoordination, we performed a series of tests, including (i) a ledge test to measure coordination, which is the symptom most directly related to signs of ataxia in patients (Video S1A–D); (ii) a hind limb clasping test, which has been used to evaluate disease progression in several mouse models of neurodegeneration (Video S2A–D); (iii) a gait test, which measures coordination and muscle function (Video S3A–D, Figure S1); and (iv) a kyphosis test, which assesses for a characteristic dorsal curvature of the spine, a common manifestation caused by a loss of muscle tone in the spinal muscles secondary to neurodegeneration (Video S4A–D). Each test is scored from 3 to 0, with 3 being the highest score in normal animals and 0 being the most severe manifestation of symptoms in tumor-bearing mice. The scores from all four tests were combined to create a composite ataxia score.

**Figure 1:**
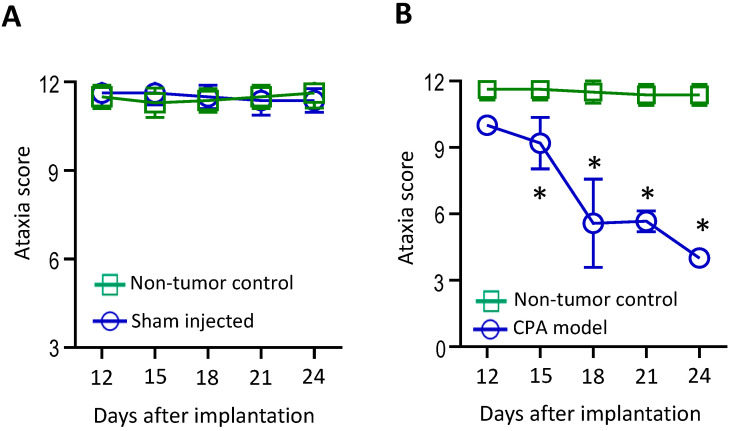
A panel of functional tests was established to evaluate the severity of ataxia symptoms in the VS mouse model. (**A**) Non-tumor-bearing normal mice without surgery or injection (control, *n* = 12) and mice that underwent unilateral sham surgery and saline injection (sham, *n* = 12) showed no differences in ataxia scores. (**B**) Compared to non-tumor-bearing mice (*n* = 12), mice bearing an *Nf2*^−/−^ tumor in the CPA model (*n* = 12) started to show a decreased ataxia score 2 weeks after tumor implantation, which deteriorated over time. Data are presented as mean ± SEM and are representative of at least three independent experiments. * *p* < 0.05. Institutional regulatory board permission was obtained for all procedures performed within this protocol from MGH IACUC.

**Figure 2:**
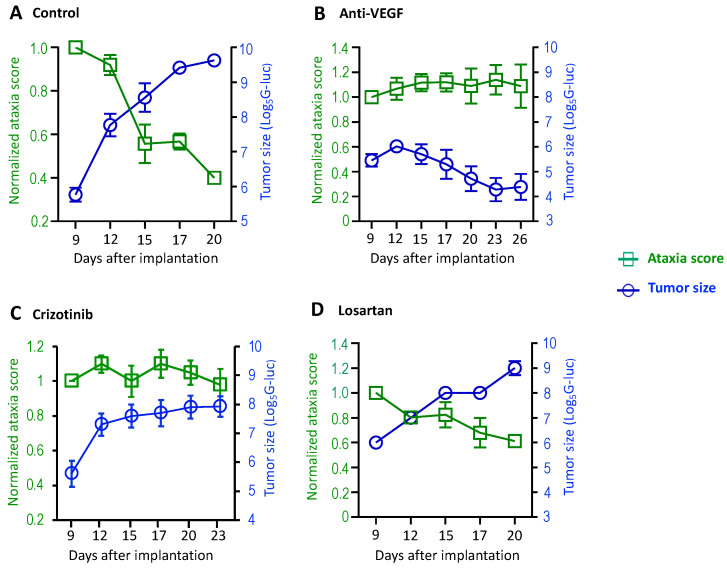
The treatment effects on vestibular symptoms were evaluated in mouse models of NF2-related VSs. *Nf2*^−/−^ tumor cells (1 × 10^5^ cells/mouse) were injected into the CPA region of the right hemisphere of mice. When the blood G-Luc level reached 2 × 10^6^ RLU, the mice were randomized to receive (**A**) isotype control IgG (*n* = 8), (**B**) anti-VEGF antibody (B20, *n* = 8), (**C**) crizotinib (*n* = 8), or (**D**) losartan (*n* = 8) treatments. Ataxia tests and tumor measurement by blood G-Luc assay were performed every 3 days until the end of the experiment. Data are presented as mean ± SEM and are representative of at least three independent experiments. Institutional regulatory board permission was obtained for all procedures performed within this protocol from MGH IACUC.

In the CPA model, the surgery and tumor implantation procedures did not result in ataxia symptoms (Figure 1A). Mice developed symptoms of ataxia two weeks after tumor implantation. The severity of ataxia increased as tumor growth progressed, and the ataxia score inversely correlated with tumor size (Figure 1A and Figure 2A).

### 3.2. Anti-VEGF Treatment Prevents Tumor-Induced Ataxia Symptoms in Mouse VS Model

Next, we characterized the treatment effects on ataxia using this panel of tests. Treatment with bevacizumab, a humanized monoclonal antibody that specifically neutralizes VEGF-A, has been documented to lead to hearing improvement or tumor shrinkage in 30–60% of NF2 patients and is approved for NF2 treatment in the United Kingdom [18]. Previously, we demonstrated that anti-VEGF treatment improves motor function by reducing neuro-edema in the sciatic nerve model [14,19,20]. Here, using the panel of the ataxia test, we further characterized how anti-VEGF affects gait and coordination. In the *Nf2*^−/−^ CPA model, groups of mice were treated with a control IgG or anti-VEGF antibody (B20, Genentech) that neutralizes mouse VEGF. Tumor growth was evaluated by measuring blood Gaussia luciferase (G-Luc) levels, and the severity of motor symptoms was evaluated every 3 days. In the control group, we observed that as tumor size increased, the ataxia score decreased, indicating that tumor growth induced more severe ataxia (Figure 2A). In mice treated with anti-VEGF antibody, *Nf2*^−/−^ tumor growth was inhibited, and the ataxia score remained unchanged over time (Figure 2B).

### 3.3. cMET Blockade Prevents Tumor-Induced Ataxia Symptoms in Mouse VS Model

cMET, the tyrosine kinase receptor for hepatocyte growth factor (HGF), is known to promote tumor progression and confer resistance to chemotherapy, radiation, and targeted therapies [21]. cMET pathway is activated in VS, and cMET inhibition triggers Schwann cell apoptosis [22,23]. Previously, we reported that cMET blockade by crizotinib (a cMET inhibitor) sensitizes VS to radiation therapy and protects hearing [8], and a phase II clinical trial using crizotinib is currently ongoing (NCT04283669) for NF2 and progressive sporadic VSs. We further evaluated how cMET blockade affects ataxia, gait, and coordination. In mice treated with crizotinib, tumor growth was stabilized and the ataxia score remained unchanged over time (Figure 2C).

### 3.4. Losartan Treatment Prevents Tumor-Induced Hearing Loss but Does Not Affect Ataxia in Mouse VS Model

Previously, we demonstrated that losartan, an FDA-approved anti-hypertensive agent that blocks angiotensin II receptor 1, prevented tumor-induced hearing loss by reducing the angiotensin inflammatory signaling in the VS model [9]. Losartan has been suggested as a possible therapy for tinnitus because losartan prevented maladaptive axonal sprouting in the cochlear nucleus after cochlear ablation in rats [24]. In the CPA model, we evaluated how losartan affects ataxia. In the group of mice treated with losartan, tumor growth was not affected, and the ataxia score decreased over time (Figure 2D).

### 3.5. Rotarod Performance Inversely Correlates with Tumor Size in VS Mouse Model

**Figure 3:**
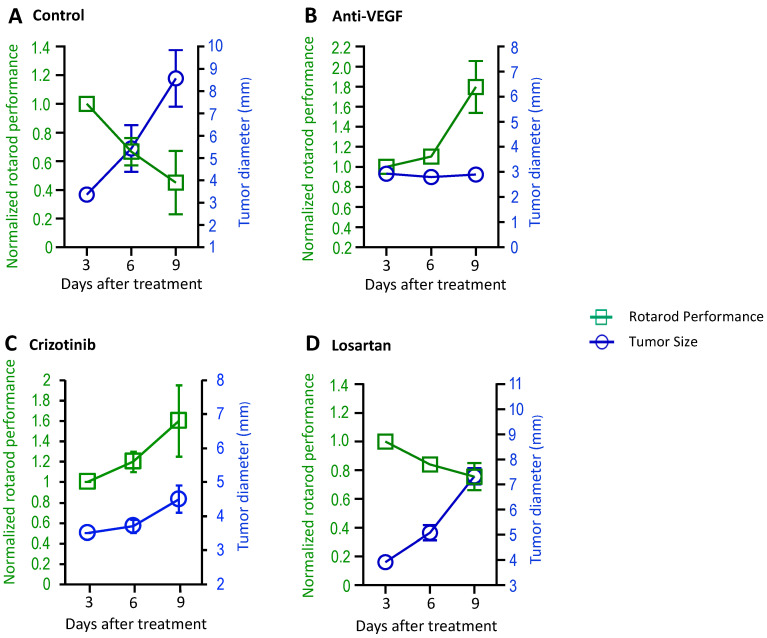
Treatment effects on motor function were evaluated in mouse models of NF2-related VS. *Nf2*^−/−^ tumor cells (1 × 10^5^ cells/mouse) were injected into the sciatic nerve of mice. When tumors reached 3–4 mm in diameter, the mice were randomized to receive (**A**) control (*n* = 7), (**B**) anti-VEGF antibody (B20, *n* = 8), (**C**) crizotinib (*n* = 8), or (**D**) losartan (*n* = 8) treatments. The rotarod performance test and tumor measurement by caliper were performed at 3 time points. Data are presented as mean ± SEM and are representative of at least three independent experiments. Institutional regulatory board permission was obtained for all procedures performed within this protocol from MGH IACUC.

Patients with VSs may also experience muscle weakness [11]. To evaluate changes in the motor function of mice, in the sciatic nerve model, we started to perform the rotarod test 3 days after treatments (Video S5A,B). In the same treatment setting as described above, the rotarod performance test and tumor measurement were performed at three time points. We found that rotarod performance decreased as tumor size increased in the control group (Figure 3A); in the anti-VEGF-treated group, where the treatments reduced tumor size, rotarod performance significantly increased (Figure 3B); in the crizotinib-treated group, where the treatment stabilized tumor growth, rotarod performance improved (Figure 3C); and in the losartan treatment groups, where treatment did not affect tumor growth, rotarod performance decreased as the tumors grew (Figure 3D).

## 4. Discussion

Patients with brain tumors face serious challenges in maintaining quality of life. Primary brain tumors originate either in the brain parenchyma (e.g., gliomas, which include astrocytomas, oligodendrogliomas, ependymomas, and medulloblastomas) or in the extraneural structures (e.g., meningiomas and schwannomas). Secondary brain tumors develop when cancer cells metastasize to the brain (e.g., lung and breast cancer brain metastasis). These patients experience general symptoms resulting from increased intracranial pressure, such as headache, anorexia, nausea, vomiting, seizures, blurred or double vision, and insomnia [3,12]. There are few well-tested interventions to improve the neurological function and quality of life among patients with brain tumors. A major limitation in studying tumor-induced neurological deficits and developing new therapeutics is the lack of orthotopic mouse models that allow for assessment of function.

To faithfully recapitulate human disease, orthotopic animal models have been developed for glioblastoma [25,26], medulloblastoma [27,28], and NF2-related vestibular schwannomas [7,29]. The majority of preclinical animal studies have primarily focused on tumor growth. However, there has been a notable shift towards investigating and addressing the impact of brain tumors on patient quality of life. In patients with NF2, although vestibular deficits caused by the tumor damaging the vestibular nerve and the vestibule have been well documented [10,11,30,31], research on characterizing and managing the vestibular dysfunction in NF2 patients is still in its infancy. Recently, Madhani et. al. evaluated vestibular-mediated behaviors (eye movements, motion perception, and balance) and clinical vestibular disability (dizziness and ataxia) in eight untreated patients with NF2-SWN and two bevacizumab-treated patients. They demonstrated that, coupled with imaging and hearing results, the vestibular tests showed VS tumors degraded vestibular precision and caused clinical disability, and bevacizumab treatment improved vestibular precision and clinical disability in both patients [12]. In the mouse model for NF2-related vestibular schwannomas, we observed that as the tumors progressed, tumor-bearing mice developed ataxia. Based on this recently published study and our observation in the mouse model, we set out to establish vestibular tests suitable for the mouse VS model and investigate how tumor growth and treatment affect gait and coordination in mouse VS models.

The first goal of our study was to identify tests that allow for rapid and sensitive quantification of gait and motor dysfunction in the mouse models. Previously published protocols have reported assessments of cerebella ataxia in several neurological disease models, including spinocerebellar ataxias, Huntington’s disease, and spinobulbar muscular atrophy [17,32,33]. Based on these published protocols, we combined five tests to evaluate tumor-induced neurological dysfunction and treatment effects. In our NF2-SWN CPA model, we found that the ledge test—a direct measure of coordination—was the most sensitive test, and changes in ledge test scores could easily be observed in the early tumor stage. The gait test that measures coordination and the kyphosis test that evaluates the characteristic dorsal curvature of the spine showed differences with late- to end-stage tumors. We used the rotarod performance test to evaluate motor function. The rotarod test is a relatively straightforward procedure and can accurately reflect motor function in both CPA and sciatic nerve models. Using this panel of five functional tests, we investigated how tumor growth affected ataxia. We showed that (i) the surgery and tumor implantation procedures used to create our animal models did not result in ataxia or incoordination and that (ii) the severity of the ataxia and incoordination worsened as the tumors grew, which mimicked the clinical disability induced by progressively enlarging VSs in patients with NF2-SWN.

Our second goal was to test for treatments that can alleviate ataxia. In the clinic, management of ataxic disorders is often very challenging [34,35]. While rehabilitation therapies help patients with ataxia, there are currently no FDA-approved treatments for ataxia [36]. Using our CPA model and the panel of ataxia tests, we investigated the potential of previously documented treatments to control tumor growth or prevent tumor-induced hearing loss in alleviating ataxia. In the clinic, treatment with bevacizumab has been documented to lead to hearing improvement or tumor shrinkage in 30–60% of patients with NF2, and it is now approved for NF2 treatment in the UK [18]. In the CPA model, using the ataxia tests we established, we demonstrated here that anti-VEGF treatment inhibited tumor growth and prevented the deterioration of ataxia. This reproduced the improvement in vestibular function observed in bevacizumab-treated VS patients [12]. Next, we tested cMET blockade, which has been reported to be a potential treatment for VSs. Previously, we demonstrated that cMET blockade by crizotinib (a cMET inhibitor) sensitizes VSs to radiation therapy and protects hearing [8], and a phase II clinical trial using crizotinib is currently ongoing (NCT04283669) for NF2 and progressive sporadic VSs. Here, we further demonstrate that cMET blockade by crizotinib treatment stabilized tumor growth in the CPA model, and as a result, it led to improvements in both ataxia and rotarod performance. Lastly, we evaluated the effects of losartan, which has been reported to prevent tumor-induced hearing loss in the VS mouse model [9] and alleviate tinnitus in a rat hearing loss model [24]. In the CPA model, treatment with losartan did not impact tumor growth and had no effects on ataxia or motor function. In addition to tumor size, edema related to the tumor is another factor contributing to the development of ataxia. Previously, we demonstrated that anti-VEGF, by normalizing the tumor vasculature, reduces perineuronal edema and improves motor function in a sciatic nerve mouse model [14]. We also reported that losartan treatment by normalizing the tumor microenvironment and inhibiting angiotensin inflammatory signaling reduces both vascular and inflammatory edema of the brain [9]. Although both treatments demonstrated a reduction in edema, only the anti-VEGF treatment, with its ability to reduce tumor volume, exhibited efficacy in ameliorating ataxia and motor function. Future studies in both preclinical mouse models and in patients are needed to characterize how edema affects ataxia and cerebellar symptoms and how edema treatment may improve these symptoms.

## 5. Conclusions

We used a panel of five tests to characterize how tumor growth and treatments affect gait, coordination, and motor function in VS models. We demonstrated that these tests can reflect the treatment effects on tumor growth on both ataxia and motor function. Our studies demonstrated that these methods, paired with hearing tests, enable a comprehensive evaluation of tumor-induced neurological deficits and facilitate the assessment of the effectiveness of novel therapeutics to improve NF2 treatments.

## Data Availability

All data reported in this study is included in the manuscript.

## Supplementary Materials

The following supporting information can be downloaded at: https://www.mdpi.com/article/10.3390/cancers16111961/s1, Figure S1. Gait analysis; Video S1. Ledge test; Video S2. Hind limb clasping test; Video S3. Gait test; Video S4. Kyphosis test; Video S5. Rotarod performance test.
