# Supplementary material for: Integrating Ataxia Evaluation into Tumor-Induced Hearing Loss Model to Comprehensively Study NF2-Related Schwannomatosis
Source: Cancers (Basel). 2024 May 22;16(11):1961. doi: 10.3390/cancers16111961 (PMC11171041; doi:10.3390/cancers16111961)
Supplement: Supplementary file 1 [file cancers-16-01961-s001.zip › Cancers-3003283_Supplementary materials.pdf]

Legends for movies

Movie 1. Ledge test. A ledge test was performed by lifting the mouse and placing it on the ledge of the cage, and by observing the mouse as it walked along the cage ledge. (a) Ledge test score 3. The mouse walked along the ledge and descended back into the cage using its paws. (b) Ledge test score 2. The mouse stayed on the ledge > 10 seconds but lost its footing and fell off the ledge. (c) Ledge test score 1. The mouse stayed on the ledge < 10 seconds and lost its footing and fell off the ledge. (d) Ledge test score 0. The mouse could not stay on the ledge for over 3 seconds, or shook and refused to move at all.

Movie 2. **Hind-limb clasping test**. Hind-limb clasping test was performed by suspending mice by the tail, and observing the movement of their forelimbs and hindlimbs into their body. (a) Hind-limb clasping test score 3. The mouse splayed out its hindlimbs. (b) Hind-limb clasping test score 2. One hindlimb was retracted to the belly. (c) Hind-limb clasping test score 1. Both hindlimbs were partially retracted. (d) Hind-limb clasping test score 0. Both of the hindlimbs were entirely retracted and touching the belly.

Movie 3. Gait test. The gait of the mouse during spontaneous walking was analyzed. (a) Gait test score 3. No limp and the belly does not touch the ground. (b) Gait test score 2. The mouse walked slowly with slight limping. (c) Gait test score 1. The mouse showed severe limping and lost coordination. (d) Gait test score 0. The mouse had difficulty moving forward, dragged its body along the ground, and could not walk a straight line.

Movie 4. Kyphosis test. The Kyphosis test was performed by assessing the characteristic dorsal curvature of the spine of the mouse. The mouse was removed from its cage and placed on a flat surface. (a) Kyphosis test score 3. The mouse straightened its spine as it walked. (b) Kyphosis test score 2. The mouse could straighten its spine and exhibited mild hunchback. (c) Kyphosis test score 1. The mouse could not straighten its spine and exhibited a mild hunchback. (d) Kyphosis test score 0. The mouse exhibited a pronounced hunchback.

Movie 5. Rotarod performance test. Mice bearing size-matched *Nf2*$^{-/-}$ tumors in the sciatic nerve, were treated with (a) control IgG, or (b) anti-VEGF (B20). Rotarod performance test was done to evaluate motor function.

Supplemental Materials

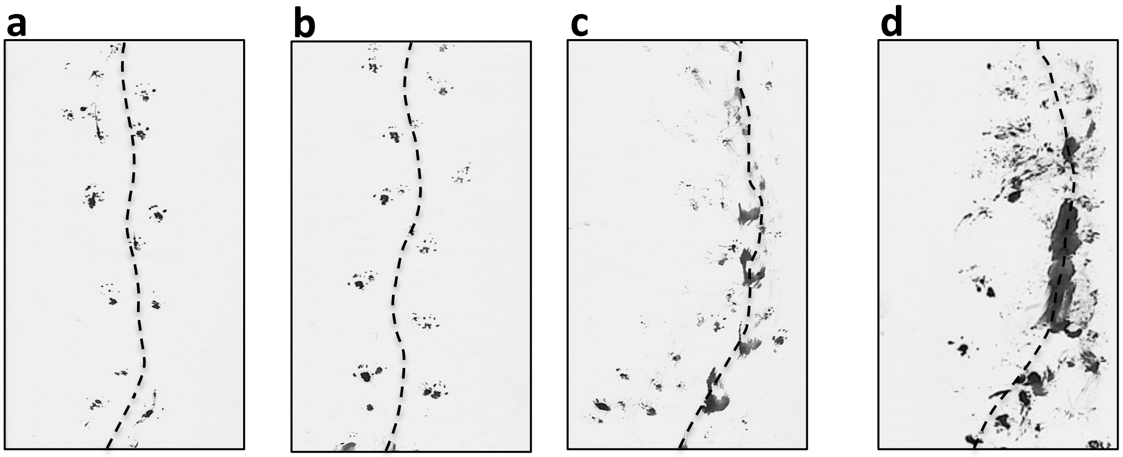

sFigure 1. Gait analysis. The footprint of the gait of the mouse. To obtain footprints, hind- and forepaws were dipped in black ink. Then, animals were allowed to walk, and footprint patterns made on white paper were obtained. (a) The footprint of the mouse with gait test score 3. The mouse walked in a straightforward manner with clear sequential steps. (b) The footprint of the mouse with gait test score 2. The mouse walked in a slightly curved pattern with clear steps; no significant footprint lag. (c) The footprint of the mouse with gait test score 1. The mouse walked with missteps (foot replacement), slight foot, and abdominal drag. (d) The footprint of the mouse with a gait test score of 0. The mouse walked with significant foot and abdominal drag.
